# Supplementary material for: An In-Depth Study of Metabolite Profile and Biological Potential of Tanacetum balsamita L. (Costmary)
Source: Plants (Basel). 2022 Dec 20;12(1):22. doi: 10.3390/plants12010022 (PMC9824382; doi:10.3390/plants12010022)
Supplement: Supplementary file 1 [file plants-12-00022-s001.zip › Suppl_material_Fig S1-4; Table S1_assays protocols.pdf]

An in-depth study of metabolite profile and biological potential of *Tanacetum balsamita* L. (costmary)

Reneta Gevrenova[1*], Gokhan Zengin[2], Kouadio Ibrahime Sinan[2], Dimitrina Zheleva-Dimitrova[1], Vessela Balabanova[1], Maxime Kolmayer[3], Yulian Voynikov[4], Olivier Joubert[3]

[1]Department of Pharmacognosy, Faculty of Pharmacy, Medical University-Sofia
[2]Department of Biology, Faculty of Science, Selcuk University, Campus, 42250, Konya, Turkey
[3]Institut Jean Lamour, UMR CNRS 7198, Université de Lorraine, CNRS, IJL, F-54000 Nancy, France
[4]Department of Chemistry, Faculty of Pharmacy, Medical University-Sofia, Bulgaria

Supplementary material

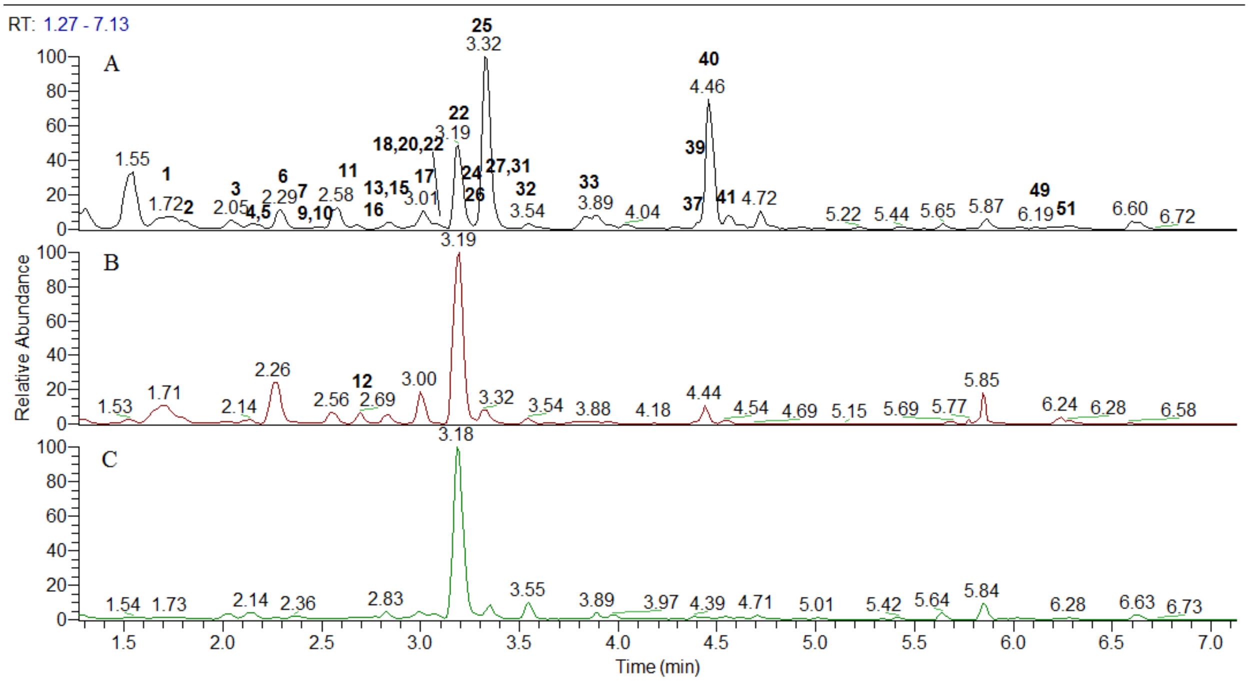

Figure S1. Extracted ion chromatogram of hydroxybenzoic and hydroxycinamic acids, and derivatives in negative ion mode of methanol-aqueous extracts from *Tanacetum balsamita* leaves (A), roots (B), flower heads (C) (for the compound numbers see Table 2).

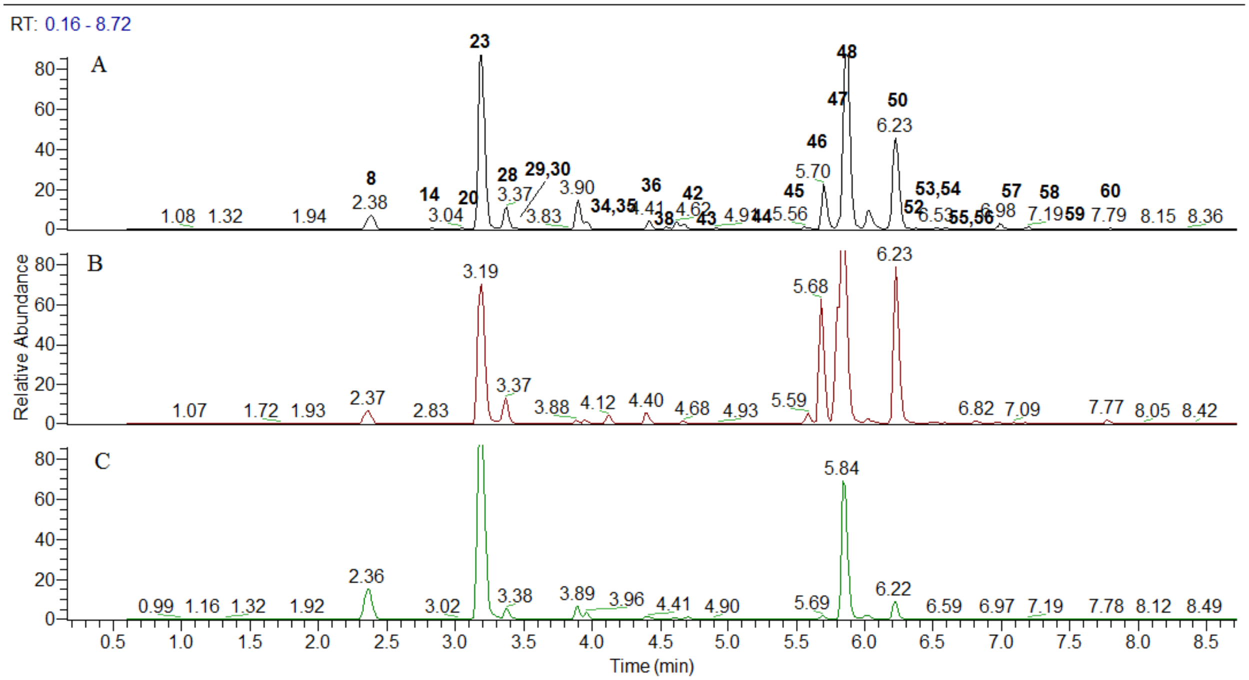

Figure S2. Extracted ion chromatogram of acylquinic acids in negative ion mode of methanol-aqueous extracts from *Tanacetum balsamita* leaves (A), roots (B), flower heads (C) (for the compound numbers see Table 2).

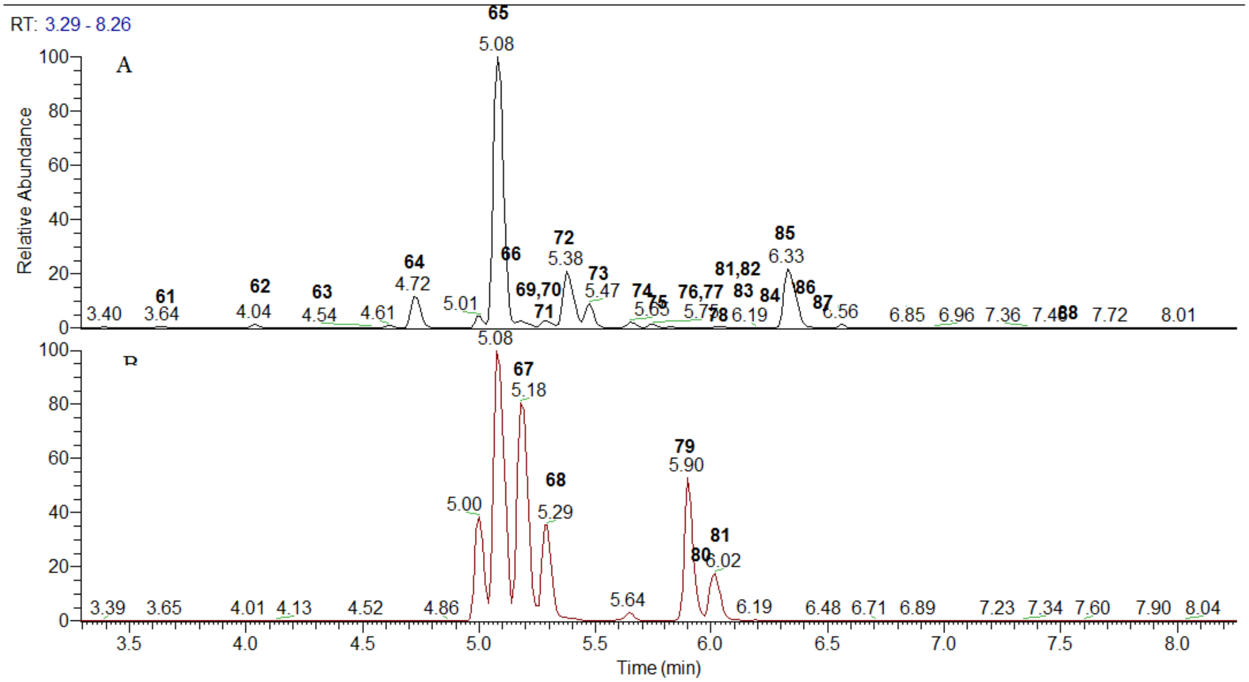

Figure S3. Extracted ion chromatogram of flavonoid glycosides in negative ion mode of methanol-aqueous extracts from *Tanacetum balsamita* leaves (A), flower heads (B) (for the compound numbers see Table 2).

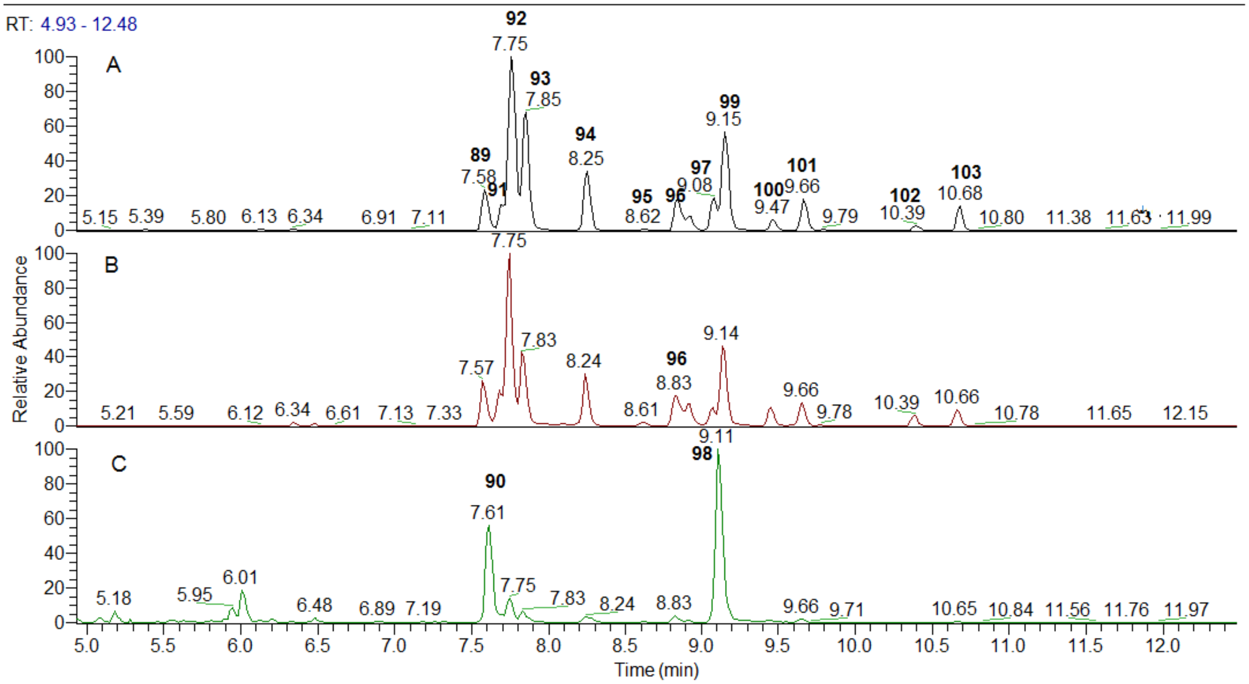

Figure S4. Extracted ion chromatogram of flavonoid aglycones in negative ion mode of methanol-aqueous extracts from *Tanacetum balsamita* leaves (A), roots (B), flower heads (C) (for the compound numbers see Table 2).

Table S1. Flavonoids in *Tanacetum balsamita* extracts assayed by UHPLC-ESI-MS/MS in positive ion mode.

No	Identified/tentatively annotated compound	Molecular formula	Exact mass $[M+H]^+$	Fragmentation pattern in (+) ESI-MS/MS	t_R (min)	Δ ppm	Distribution
**Flavonoids**							
**61.**	naringenin 6, 8-di*C*-hexoside	$C_{27}H_{32}O_{15}$	597.1814	597.1808 Not fragmented	3.64	-1.233	1,2
**62.**	apigenin-6, 8-di*C*-hexoside[b]	$C_{27}H_{30}O_{15}$	595.1657	595.1643 (100), 577.1537 (59.3), 559.1413 (17.2), 529.1317 (8.7), 511.1219 (5.4), 499.1234 (6.2), 487.1017 (2.9), 475.1234 (15.7), 439.1035 (9.7), 457.1113 (31.5), 427.0966 (11.0), 409.0892 (17.7), 379.0807 (34.6), 361.0687 (8.4), 355.0806 (13.7), 325.0700 (45.6), 295.0593 (41.7), 283.0597 (3.1), 268.0741 (3.7), 177.0179 (2.6), 121.0284 (22.5)	4.04	2.414	2
**63.**	homoorientin (luteolin-6-*C*-glucoside)[a]	$C_{21}H_{20}O_{11}$	449.1078	449.1068 (93.4), 431.0966 (40.8), 413.0862 (17.9), 395.0754 (18.3), 383.0752 (21.5), 353.0649 (45.3), 329.0649 (73.0), 311.0548 (9.8), 299.0546 (100), 283.0594 (8.1), 165.0182 (26.7), 137.0232 (10.4)	4.54	-2.355	1, 2,3
**64.**	luteolin-*O*-hexuronosyl-*O*-hexoside	$C_{27}H_{28}O_{17}$	625.1399	625.1385 (31.8), 463.0851 (6.0), 287.0544 (100), 241.0489 (0.6), 153.0182 (7.2), 137.0229 (0.5), 135.0439)	4.72	-2.281	2
**65.**	rutin[a,b]	$C_{27}H_{30}O_{16}$	611.1607	611.1566 (0.8), 465.1016 (9.5), 303.0495 (100), 285.0388 (0.6), 257.0440 (2.5), 247.0607 (0.5), 229.0495 (2.9), 165.0180 (0.8), 153.0183 (3.8), 137.0235 (3.2)	5.08	-6.645	1,2
**66.**	luteolin-*O*-pentosyl-*O*-hexoside	$C_{26}H_{28}O_{15}$	581.1501	581.1501 (15.9), 449.1070 (21.7), 287.0544 (100), 241.0490 (0.3), 153.0181 (5.4), 137.0234 (0.7), 135.0443 (1.9)	5.09	-2.437	2
**67.**	isoquercitrin	$C_{21}H_{20}O_{12}$	465.1028	465.1022 (2.5), 303.0495 (100), 285.0380 (0.6), 274.0459 (0.5), 257.0429 (1.9), 229.0499 (3.5), 165.0180 (2.0), 137.0236 (4.0), 153.0183 (3.9),	5.18	0.059	1,2,3
**68.**	hyperoside	$C_{21}H_{20}O_{12}$	465.1028	465.1028 (5.6), 303.0495 (100), 285.0379 (0.5), 274.0461 (0.3), 257.0439 (1.5), 229.0490 (2.8), 165.0182 (1.4), 137.0235 (2.6), 153.0182 (8.0),	5.29	-1.123	1,2,3
**69.**	nepetin-pentosylhexoside	$C_{27}H_{30}O_{16}$	611.1607	611.1575 (21.5), 479.1175 (22.3), 317.0649 (100), 302.0415 (49.2), 272.0328 (9.7), 168.0053 (9.2), 137.0231 (1.5), 153.0176 (0.6)	5.35	-5.123	1,2

**70.**	luteolin-7-*O*- rutinoside[b]	$C_{27}H_{30}O_{15}$	595.1657	595.1645 (15.4), 449.1068 (28.3), 287.0544 (100), 153.0181 (5.4), 135.0439 (1.2)	5.22	-2.094	1,2
**71.**	luteolin 7-*O*-glucoside[a]	$C_{21}H_{20}O_{11}$	449.1078	449.1066 (10.9), 287.0543 (100), 269.0439 (2.3), 241.0498 (0.3), 153.0181 (0.9), 121.0284 (1.1)	5.31	-2.756	1, 2,3
**72.**	luteolin-*O*-hexuronide	$C_{21}H_{18}O_{12}$	463.0871	463.0857 (28.2), 287.0544 (100), 269.0438 (2.2), 241.0490 (0.9), 213.0525 (0.3)	5.38	2.985	2
**73.**	isorhamnetin-*O*-hexuronide	$C_{22}H_{20}O_{13}$	493.0977	493.0967 (49.0), 317.0651 (100), 302.0416 (36.9), 274.0467 (27.1), 153.0181 (0.6)	5.47	-1.941	2
**74.**	kaempferol-7-*O*-rutinoside	$C_{27}H_{30}O_{15}$	595.1657	595.1620 (0.7), 449.1076 (11.4), 287.0544 (100), 258.0505 (0.7), 165.0178 (0.7), 153.0182 (2.5), 121.0286 (2.3)	5.65	-6.295	1,2
**75.**	nepetin-*O*-hexoside	$C_{22}H_{22}O_{12}$	479.1184	479.1164 (14.6), 317.0650 (100), 302.0415 (43.6), 135.0444 (0.5), 137.0235 (2.2)	5.67	4.200	1,2,3
**76.**	Axillarin-*O*-pentosylhexoside	$C_{28}H_{32}O_{17}$	641.1712	641.1666 (19.2), 509.1274 (31.5), 347.0755 (100), 331.0453 (14.0), 289.0460 (12.8), 269.0435 (5.1)	5.74	-7.168	2
**77.**	apigenin-*O*-pentosylhexoside	$C_{26}H_{28}O_{14}$	565.1552	565.1533 (15.4), 433.1121 (25.2), 271.0597 (100), 243.0640 (0.4), 225.0541 (0.4), 163.0387 (0.6), 153.0181 (4.2), 121.0287 (0.7), 119.0494 (1.7)	5.75	-3.294	2
**78.**	apigenin-*O*-rutinoside	$C_{27}H_{30}O_{14}$	579.1708	579.1693 (13.6), 433.1123 (25.5), 271.0596 (100), 153.0182 (5.1), 163.0387 (0.3), 121.0286 (0.6), 119.0496 (2.0)	5.82	-2.645	2
**79.**	isorhamnetin 3-*O*-glucoside[a,b]	$C_{22}H_{22}O_{12}$	479.1184	479.1188 (1.4), 317.0649 (100), 302.0415 (6.5), 274.0463 (3.9), 246.0512 (1.8), 229.0493 (3.3), 165.0182 (0.3), 153.0181 (7.4)	5.90	-0.005	1,2,3
**80.**	hispidulin *O*-pentosylhexoside	$C_{27}H_{30}O_{15}$	595.1657	595.1642 (23.8), 463.1221 (31.2), 301.0701 (100), 286.0467 (40.0), 258.0514 (11.5)	5.93	-2.632	2
**81.**	isorhamnetin-*O*-pentoside	$C_{21}H_{19}O_{11}$	449.1078	449.1071 not fragmented	6.02	-1.598	1, 2
**82.**	chrysoeriol O-pentosylhexoside	$C_{27}H_{30}O_{15}$	595.1657	595.1640 (14.5), 463.1209 (15.2), 301.0703 (79.7), 286.0468 (39.9), 255.0646 (4.4), 151.0388 (28.9), 149.0234 (35.0)	6.04	-2.935	2
**83.**	apigenin-*O*-hexuronide[b]	$C_{21}H_{18}O_{11}$	447.0922	447.0912 (27.5), 271.0596 (100), 253.0495 (0.2), 243.0644 (0.4), 225.0546 (0.4), 163.0392 (0.5), 153.0181 (6.5), 121.0286 (0.9), 119.0494 (2.6)	6.13	-2.232	2
**84.**	kaempferol-3-*O*-glucoside[a]	$C_{21}H_{20}O_{11}$	449.1078	449.1052 (25.3), 287.0544 (100), 153.0182 (2.1)	6.21	-5.896	1, 2
**85.**	jaceosidin-*O*-hexuronide[b]	$C_{23}H_{22}O_{13}$	507.1133	507.1126 (47.5), 331.0806 (100), 315.0493 (39.2), 301.0339 (2.9), 287.0542 (4.6), 273.0392 (1.6), 258.0518 (2.4), 153.0181 (0.5)	6.33	-2.731	2,3

**86.**	chrysoeriol-*O*-hexuronide[b]	$C_{22}H_{20}O_{12}$	477.1028	477.1019 (44.3) 301.0701 (100), 286.0466 (42.0), 258.0518 (22.4), 229.0487 (0.5),153.0185 (0.5),	6.34	-1.850	2
**87.**	jaceosidin *O*-hexoside	$C_{23}H_{24}O_{12}$	493.1341	493.1328 (19.8), 331.0805 (100), 316.0573 (35.4), 301.0337 (0.6), 273.0381 (6.3), 245.0432 (2.), 151.0391 (0.9), 168.0051 (8.4)	6.50	-2.459	2
**88.**	eupatilin O-hexoside	$C_{24}H_{26}O_{12}$	507.1497	507.1483 (19.4), 345.0962 (100), 330.0724 (27.1), 315.0489 (2.4), 287.0533 (1.5), 269.0444 (0.3), 259.0591 (0.3), 168.0053 (6.6), 165.0548 (1.3)	7.49	-2.667	2
**89.**	luteolin[a]	$C_{15}H_{10}O_6$	287.0550	287.0544 (100), 269.0435 (0.8), 241.0490 (0.9), 231.0660 (0.1), 213.0550 (0.3), 203.0340 (0.2), 153.0182 (10.5), 137.0234 (1.5), 135.0441 (3.6),	7.58	-2.106	2,3
**90.**	quercetin[a]	$C_{15}H_{10}O_7$	303.0499	303.0492 (100), 285.1217 (5.5), 257.0446 (1.7), 229.0493 (8.2), 153.0183 (7.9), 137.0233 (5.9)	7.63	-2.538	1,2
**91.**	patuletin (6-methoxyquercetin)	$C_{16}H_{12}O_8$	333.0605	333.0597 (100), 318.0364 (28.1), 273.0381 (2.7), 168.0051 (0.7), 137.0235 (3.3),	7.72	-2.413	2,3
**92.**	nepetin (6-methoxyluteolin)	$C_{16}H_{12}O_7$	317.0656	317.0651 (100), 302.0418 (17.5), 274.0467 (30.8), 203.0336 (0.8), 153.0180 (0.9), 178.0869 (2.9), 132.0809 (3.5)	7.75	-1.637	1,2,3
**93.**	spinacetin	$C_{17}H_{14}O_8$	347.0761	347.0755 (100), 332.0518 (31.6), 317.0289 (4.8), 289.0338 (7.6), 261.0391 (5.4), 233.0438 (0.5), 168.0051 (8.4)	7.85	-1.942	2,3
**94.**	axillarin	$C_{17}H_{14}O_8$	347.0761	347.0738 (100), 332.0518 (13.5), 317.0293 (2.7), 289.0336 (11.7), 168.0052 (1.6), 137.0233 (1.2)	8.25	-0.205<br>-1.509	2,3
**95.**	apigenin[a]	$C_{15}H_{10}O_5$	271.0601	271.0597 (100), 243.0635 (0.5), 229.0477 (0.3), 163.0383 (0.7), 153.0182 (10.1), 121.0285 (1.4), 119.0493 (5.5)	8.62	8.61	2
**96.**	hispidulin (scutellarein-6-methyl ether)[a]	$C_{16}H_{12}O_6$	301.0707	301.0700 (100), 286.0465 (24.0), 258.0517 (28.7), 229.0494 (1.9), 168.0050 (1.1), 121.0295 (0.4),	8.84	-0.372<br>-2.075	2,3
**97.**	quercetagetin-3,6,3'(4')-trimethyl ether	$C_{18}H_{16}O_8$	361.0918	361.0915 (100), 346.0676 (30.3), 331.0446 (1.4), 316.0229 (0.2), 257.0435 (2.1), 229.0493 (11.9), 168.0052 (7.0), 163.0385 (0.9), 139.0028 (0.3)	9.08	1.112	2,3
**98.**	isorhamnetin[a]	$C_{16}H_{12}O_7$	317.0656	317.0650 (100), 302.0430 (4.2), 153.0182 (6.5)	9.11	-1.827	1,2
**99.**	jaceosidin (6-hydroxyluteolin-6,3'-dimethyl ether)[a,b]	$C_{17}H_{14}O_7$	331.0812	331.0803 (100), 316.0570 (35.6), 301.0335 (7.4), 273.0388 (9.1), 245.0439 (6.7), 168.0051 (12.1)	9.15	-2.746	2,3
**100.**	cirsiliol	$C_{17}H_{14}O_7$	331.0803	331.0806 (100), 315.0493 31.10), 301.0337 (1.1), 273.0389 (1.6), 245.0435 (0.1), 163.0179 (1.1),	9.47	-1.901	2,3

				137.0218 (0.1), 136.0147 (0.2)			
**101.**	quercetagetin-3,6,3'(4')-trimethyl ether	$C_{18}H_{16}O_8$	361.0918	361.0909 (100), 346.0675 (24.3), 331.0426 (2.4), 316.0219 (0.3), 257.0440 (3.1), 229.0493 (18.3), 169.0131 (5.3), 137.0226 (0.5), 119.0493 (0.4)	9.66	-2.448	2,3
**102.**	cirsimaritin (6-hydroxyapigenin-6,7-dimethyl ether)a,[b]	$C_{17}H_{14}O_6$	315.0863	315.0857 (100), 299.0546 (29.3), 285.0388 (1.4), 271.0597 (9.0), 242.0569 (2.4), 153.0182 (0.6)	10.39	-1.887	2,3
**103.**	eupatilin/santin	$C_{18}H_{16}O_7$	345.0969	345.0962 (100), 330.0727 (32.0), 315.0493 (2.0), 287.0537 (2.3), 259.0592 (0.7), 241.0499 (0.6), 229.0485 (0.2), 168.0052 (8.6)	10.68	-1.911	2,3

[a]-compare to reference

Assays for Total Phenolic and Flavonoid Contents

The total phenolic content was determined by employing the methods given in the literature with some modification. Sample solution (0.25 mL) was mixed with diluted Folin–Ciocalteu reagent (1 mL, 1:9, v/v) and shaken vigorously. After 3 min, Na_2CO_3 solution (0.75 mL, 1%) was added and the sample absorbance was read at 760 nm after a 2 h incubation at room temperature. The total phenolic content was expressed as milligrams of gallic acid equivalents (mg GAE/g extract)[62].

The total flavonoid content was determined using the $AlCl_3$ method. Briefly, sample solution (1 mL) was mixed with the same volume of aluminum trichloride (2%) in methanol. Similarly, a blank was prepared by adding sample solution (1 mL) to methanol (1 mL) without $AlCl_3$. The sample and blank absorbances were read at 415 nm after a 10 min incubation at room temperature. The absorbance of the blank was subtracted from that of the sample. Rutin was used as a reference standard and the total flavonoid content was expressed as milligrams of rutin equivalents (mg RE/g extract) [62].

Determination of Antioxidant and Enzyme Inhibitory Effects

Antioxidant (DPPH and ABTS radical scavenging, reducing power (CUPRAC and FRAP), phosphomolybdenum and metal chelating (ferrozine method)) and enzyme inhibitory activities (cholinesterase (Elmann's method), tyrosinase (dopachrome method), α-amylase (iodine/potassium iodide method), α -glucosidase (chromogenic PNPG method) and pancreatic lipase (*p*-nitrophenyl butyrate (p-NPB) method) were determined using the methods previously described by Uysal et al. [62] and Grochowski et al. [63].

For the DPPH (1,1-diphenyl-2-picrylhydrazyl) radical scavenging assay: Sample solution was added to 4 mL of a 0.004% methanol solution of DPPH. The sample absorbance was read at 517 nm after a 30 min incubation at room temperature in the dark. DPPH radical scavenging activity was expressed as milligrams of trolox equivalents (mg TE/g extract).

For ABTS (2,2′-azino-bis(3-ethylbenzothiazoline) 6-sulfonic acid) radical scavenging assay: Briefly, ABTS+ was produced directly by reacting 7 mM ABTS solution with 2.45 mM potassium persulfate and allowing the mixture to stand for 12–16 in the dark at room temperature. Prior to beginning the assay, ABTS solution was diluted with methanol to an absorbance of 0.700 ± 0.02 at 734 nm. Sample solution was added to ABTS solution (2 mL) and mixed. The sample absorbance was read at 734 nm after a 30 min incubation at room temperature. The ABTS radical scavenging activity was expressed as milligrams of trolox equivalents (mg TE/g extract).

For CUPRAC (cupric ion reducing activity) activity assay: Sample solution was added to premixed reaction mixture containing $CuCl_2$ (1 mL, 10 mM), neocuproine (1 mL, 7.5 mM) and NH_4Ac buffer (1 mL, 1 M, pH 7.0). Similarly, a blank was prepared by adding sample solution (0.5 mL) to premixed reaction mixture (3 mL) without $CuCl_2$. Then, the sample and blank absorbances were read at 450 nm after a 30 min incubation at room temperature. The absorbance of the blank was subtracted from that of the sample. CUPRAC activity was expressed as milligrams of trolox equivalents (mg TE/g extract).

For FRAP (ferric reducing antioxidant power) activity assay: Sample solution was added to premixed FRAP reagent (2 mL) containing acetate buffer (0.3 M, pH 3.6), 2,4,6-tris(2-pyridyl)-S-triazine (TPTZ) (10 mM) in 40 mM HCl and ferric chloride (20 mM) in a ratio of 10:1:1 (v/v/v). Then, the sample absorbance was read at 593 nm after a 30 min incubation at room temperature. FRAP activity was expressed as milligrams of trolox equivalents (mg TE/g extract).

For phosphomolybdenum method: Sample solution was combined with 3 mL of reagent solution (0.6 M sulfuric acid, 28 mM sodium phosphate and 4 mM ammonium molybdate). The sample absorbance was read at 695 nm after a 90 min incubation at 95 °C. The total antioxidant capacity was expressed as millimoles of trolox equivalents (mmol TE/g extract).

For metal chelating activity assay: Briefly, sample solution was added to $FeCl_2$ solution (0.05 mL, 2 mM). The reaction was initiated by the addition of 5 mM ferrozine (0.2 mL). Similarly, a blank was prepared by adding sample solution (2 mL) to $FeCl_2$ solution (0.05 mL, 2 mM) and water (0.2 mL) without ferrozine.

Then, the sample and blank absorbances were read at 562 nm after 10 min incubation at room temperature. The absorbance of the blank was sub-tracted from that of the sample. The metal chelating activity was expressed as milligrams of EDTA (disodium edetate) equivalents (mg EDTAE/g extract).

For Cholinesterase (ChE) inhibitory activity assay: Sample solution (was mixed with DTNB (5,5-dithio-bis(2-nitrobenzoic) acid, Sigma, St. Louis, MO, USA) (125 μL) and AChE (acetylcholines-terase (Electric ell acetylcholinesterase, Type-VI-S, EC 3.1.1.7,Sigma)), or BChE (butyrylcholinesterase (horse serum butyrylcholinesterase, EC 3.1.1.8, Sigma)) solution (25 μL) in Tris–HCl buffer (pH 8.0) in a 96-well microplate and incubated for 15 min at 25 °C. The reaction was then initiated with the addition of acetylthiocholine iodide (ATCI, Sigma) or butyrylthiocholine chloride (BTCl, Sigma) (25 μL). Similarly, a blank was prepared by adding sample solution to all reaction reagents without enzyme (AChE or BChE) solution. The sample and blank absorbances were read at 405 nm after 10 min incubation at 25 °C. The absorbance of the blank was subtracted from that of the sample and the cholinesterase inhibitory activity was expressed as galanthamine equivalents (mgGALAE/g extract).

For Tyrosinase inhibitory activity assay: Sample solution was mixed with tyrosinase solution (40 μL, Sigma) and phosphate buffer (100 μL, pH 6.8) in a 96-well microplate and incubated for 15 min at 25 °C. The reaction was then initiated with the addition of L-DOPA (40 μL, Sigma). Similarly, a blank was prepared by adding sample solution to all reaction reagents without enzyme (tyrosinase) solution. The sample and blank absorbances were read at 492 nm after a 10 min incubation at 25 °C. The absorbance of the blank was subtracted from that of the

sample and the tyrosinase inhibitory activity was expressed as kojic acid equivalents (mgKAE/g extract).

For α-amylase inhibitory activity assay: Sample solution was mixed with α-amylase solution (ex-porcine pancreas, EC 3.2.1.1, Sigma) (50 μL) in phosphate buffer (pH 6.9 with 6 mM sodium chloride) in a 96-well microplate and incubated for 10 min at 37 °C. After pre-incubation, the reaction was initiated with the addition of starch solution (50 μL, 0.05%). Similarly, a blank was prepared by adding sample solution to all reaction reagents without enzyme (α-amylase) solution. The reaction mixture was incubated 10 min at 37 °C. The reaction was then stopped with the addition of HCl (25 μL, 1 M). This was followed by addition of the iodine-potassium iodide solution (100 μL). The sample and blank absorbances were read at 630 nm. The absorbance of the blank was subtracted from that of the sample and the α-amylase inhibitory activity was expressed as acarbose equivalents (mmol ACE/g extract).

For α-glucosidase inhibitory activity assay: Sample solution was mixed with glutathione (50 μL), α-glucosidase solution (from Saccharomyces cerevisiae, EC 3.2.1.20, Sigma) (50 μL) in phosphate buffer (pH 6.8) and PNPG (4-N-trophenyl-α-D-glucopyranoside, Sigma) (50 μL) in a 96-well microplate and incubated for 15 min at 37 °C. Similarly, a blank was prepared by adding sample solution to all reaction reagents without enzyme (α-glucosidase) solution. The reaction was then stopped with the addition of sodium carbonate (50 μL, 0.2 M). The sample and blank absorbances were read at 400 nm. The absorbance of the blank was subtracted from that of the sample and the α-glucosidase inhibitory activity was expressed as acarbose equivalents (mmol ACE/g extract).
